# Efflux transporters in rat placenta and developing brain: transcriptomic and functional response to paracetamol

**DOI:** 10.1038/s41598-021-99139-6

**Published:** 2021-10-06

**Authors:** L. M. Koehn, Y. Huang, M. D. Habgood, S. Nie, S. Y. Chiou, R. B. Banati, K. M. Dziegielewska, N. R. Saunders

**Affiliations:** 1 Department of Biochemistry & Pharmacology, University of Melbourne, Parkville, VIC 3010 Australia; 2 Department of Neuroscience, Monash University, Melbourne, VIC 3004 Australia; 3 Melbourne Mass Spectrometry and Proteomics Facility, Bio21 Molecular Science and Biotechnology Institute, University of Melbourne, Parkville, VIC 3010 Australia; 4 ANSTO – Australia’s Nuclear Science and Technology Organisation, New Illawarra Rd, Lucas Heights, NSW 2234 Australia; 5 University of Sydney, Camperdown, Sydney, Australia

**Keywords:** Computational biology and bioinformatics, Developmental biology, Molecular biology, Neuroscience, Physiology, Diseases

## Abstract

Adenosine triphosphate binding cassette (ABC) transporters transfer lipid-soluble molecules across cellular interfaces either directly or after enzymatic metabolism. RNAseq analysis identified transcripts for ABC transporters and enzymes in rat E19, P5 and adult brain and choroid plexus and E19 placenta. Their functional capacity to efflux small molecules was studied by quantitative analysis of paracetamol (acetaminophen) and its metabolites using liquid scintillation counting, autoradiography and ultra-performance liquid chromatography coupled with tandem mass spectrometry (UPLC-MS/MS). Animals were treated acutely (30 min) and chronically (5 days, twice daily) with paracetamol (15 mg/kg) to investigate ability of brain and placenta barriers to regulate ABC transport functionality during extended treatment. Results indicated that transcripts of many efflux-associated ABC transporters were higher in adult brain and choroid plexus than at earlier ages. Chronic treatment upregulated certain transcripts only in adult brain and altered concentrations of paracetamol metabolites in circulation of pregnant dams. Combination of changes to metabolites and transport system transcripts may explain observed changes in paracetamol entry into adult and fetal brains. Analysis of lower paracetamol dosing (3.75 mg/kg) indicated dose-dependent changes in paracetamol metabolism. Transcripts of ABC transporters and enzymes at key barriers responsible for molecular transport into the developing brain showed alterations in paracetamol pharmacokinetics in pregnancy following different treatment regimens.

## Introduction

Paracetamol (acetaminophen) is commonly taken by prescription or self-medication for the relief of pain and to reduce fever. It is the most widely used drug in pregnancy^1^ but it is unclear what mechanisms in the placenta influence its transfer from the maternal circulation into the fetus and its brain. We have shown in earlier work that there is a marked difference in the level of paracetamol that enters the fetal rat brain at embryonic day, E19 compared to the early postnatal period (postnatal day, P4) or in the adult^2^. This study also showed that the protection provided by the placenta might be greater than that of the brain barriers. At E19 the fetal/maternal plasma ratio for paracetamol was about 40% for both acute (single dose) and chronic (5 days) treatment, indicating that the placental barrier kept about 60% of the drug in maternal blood from reaching the fetal circulation. Fetal brain/plasma and CSF/plasma ratios were about 60% with acute treatment, which increased to > 100% and 90%, respectively with chronic treatment. These last values indicate that the fetal brain barriers provided little protection against the paracetamol that enters fetal circulation over extended periods of time, at least in concentrations used in the study^2^. Subsequently we have shown that paracetamol administered to pregnant rats may provoke an inflammatory response in the placenta itself^3^. It is therefore of interest to define the mechanisms that control its transfer across placenta and brain barriers as this could provide a basis for developing methods to limit paracetamol entry across the placenta thus limiting its potential harmful effects on the developing fetal brain. Despite being widely considered as ‘safe’ for use even in pregnancy^4^ numerous studies have suggested that fetal exposure to paracetamol could have long-term neurological implications, although recent reviews have drawn attention to significant methodological limitations in these studies e.g.^5–7^.

Mechanisms influencing paracetamol entry into the brain are likely to involve ABC efflux transporters or their related enzyme systems^8–10^. The aim of the present study was to use Illumina RNA-Sequencing (RNA-Seq) of E19 placenta and fetal, early postnatal (P5) and adult brain and choroid plexus to determine the level of transcriptomic expression of these transporters and their related enzymes in these tissues. We also tested their regulatory response to paracetamol exposure. In functional studies the concentration and transfer ratios of paracetamol (and its metabolites) were measured across placental and brain barriers at two different drug doses (3.75 or 15 mg/kg) and durations of administration (acute or 5 days). These experiments were carried out to to investigate if varying exposure to paracetamol could influence the effectiveness of brain and placenta barriers to this drug in vivo.

## Methods

### Ethics statement

The animal model used in this study was the Sprague Dawley strain of *rattus norvegicus*. All animal experimentation was approved by the University of Melbourne Animal Ethics Committee (Ethics Permission AEC: 1714344.1) and conducted in compliance with Australian National Health and Medical Research Guidelines and ARRIVE guidelines. All animals were assessed as healthy prior to commencement of experiments. Animals were monitored prior to and following every injection ensuring there were no abnormalities in weight (> 10%), appearance (fur) or behaviour (vocalisation, respiration, movements). All efforts were made to ameliorate any suffering of animals. They were handled by experienced researchers in such a way as to minimise stress prior to being terminally anaesthetised.

### Animals

The rats were supplied by the University of Melbourne Biological Research Facility and subjected to a 12 h light/dark cycle with ad libitum access to water and food (dry pellets of a fixed formulation diet for laboratory rats and mice fortified with vitamins and minerals to meet the requirements of breeding animals after the diet is autoclaved or irradiated, as supplied by Specialty Feeds, Western Australia). Animals were housed in groups of 2–4 (adult) per cage (25 cm × 35 cm × 25 cm on Breeders Choice paper bedding, made from 99% recycled paper; it is biodegradable with no added chemicals). Age groups investigated (at treatment completion) were embryonic day 19 (E19) pups (n = 104) and dams (n = 15), which were all primigravida (350–400 g body weight at termination of experiments), P4–5 neonates (dependent on precise birth time overnight, n = 24) and non-pregnant adults (175–230 g body weight, n = 19). The numbers (n) of animals used for each experiment are indicated in the relevant Methods or Results section and where appropriate in legends.

E19, rather than an earlier age was chosen because this is a stage of development when adequate volumes of blood and CSF can be obtained for analysis from fetal rats without pooling^11^. Dating of animals was based on taking E0 as the day when a plug was identified and P0 as the day of birth.

Where possible equal numbers of male and female fetuses were used. Animals were randomly allocated to experiments by animal house staff, who had no knowledge of the particular experiments to be performed and were selected by an independent person for treatment groups to ensure body weights were statistically similar between the groups that were being compared.

### Drugs and markers

Paracetamol (acetaminophen ≥ 99.0%, Sigma-Aldrich) was administered at recommended clinical doses of 15 mg/kg, which is 80% of the highest single dose recommended for adults with severe pain and designated here as a “high dose”^4^ or 3.75 mg/kg which is 80% of the maximum tablet size authorized by the FDA for human use (https://www.fda.gov/drugs/drug-safety-and-availability/fda-drug-safety-communication-prescription-acetaminophen-products-be-limited-325-mg-dosage-unit) here designated as a “low dose” (see also^2^). Plasma measurements confirmed that the “high dose” resulted in circulating levels comparable to those identified in humans (see “Results” section). This was prioritized over selection of dose equivalents for similar nociceptive effects between humans and rats, which was not an aim of the present study. Paracetamol was dissolved in sterile 0.9% sodium chloride solution for injection. For liquid scintillation counting studies the final paracetamol injection was spiked with [2,6-^3^H]-labelled paracetamol (American Radiochemicals Inc., ART0679) 0.5–20 µCi depending on the age (size) of the animals.

### Experimental procedures

#### All experiments took place between 09.00 and 15.00 h

##### RNA-sequencing developmental study

E19 fetuses, postnatal (P5) pups, pregnant females, non-pregnant females and males were used. The E19 pregnant dams were terminally anesthetized (i.p. 25% w/v urethane, Sigma, 1 mL per 100 g body weight) and the uterus exposed by a midline abdominal excision. Urethane was selected to anaesthetize and immobilize the dam while maintaining blood flow to the fetuses during the collection period^12^. Fetuses were removed from the uterus, exsanguinated, placenta, brain and choroid plexus dissected out, flash frozen in liquid N_2_ and then stored at − 80 °C until RNA was extracted. Placental tissue was sampled as a cross section of the chorio-allantoic placental disc, following removal of the externally attached umbilical and maternal circulatory vessels. Brain was sampled as cortical tissue as previously described^12^ and choroid plexus was sampled from lateral ventricles. The postnatal and non-pregnant adult animals were terminally anaesthetized using inhaled isoflurane (IsoFlo, 100% w/w Abbott Laboratories, reduced to a lethal dose of 15% by the vaporiser) and samples taken and stored as for E19 animals.

##### RNA-sequencing paracetamol study

E19 dams were given a single i.p injection of 15 mg/kg paracetamol (dissolved in sterile 0.9% sodium chloride solution) and referred to as “acute”. For “chronic” experiments E15 pregnant dams were injected i.p. twice daily with either 3.75 mg/kg or 15 mg/kg paracetamol over 4 days. On the fifth day (E19) the dam was given a final injection of the drug, terminally anesthetized and samples taken 30 min after final injection in the same manner as described for the *developmental study.*

### Permeability studies

Time-mated E15 pregnant dams were injected i.p. twice daily with “low dose” or “high dose” paracetamol (dissolved in sterile 0.9% sodium chloride solution) over four days. On the fifth day the pregnant dams (E19) were terminally anaesthetised i.p. with 25% w/v urethane, (Sigma, 1 mL per 100 g body weight) and placed supine on a 35 °C heating plate and an endotracheal cannula inserted prior to sampling. Left femoral artery and vein were cannulated. The final injection was by slow infusion to the femoral vein; the cannula was flushed with 0.5 mL of heparinized (Hospira Inc, 5000 units per mL) saline. Maternal blood samples taken from the femoral artery were time matched to collection of individual fetal samples; blood volume was maintained by intraarterial injection of equivalent volumes of heparinized sodium chloride solution. Blood (right cardiac ventricle), cerebrospinal fluid CSF (*cisterna magna*) and brains (cortex) were sampled from each fetus. Sampling was concluded when the state of the placental circulation (normal condition: umbilical veins pink with oxygenated blood) was deemed insufficient, usually around 90 min (see^2^ for details). CSF samples were examined microscopically for traces of red blood cells and discarded if contaminated^13^. Maternal blood was also collected at the end of the experiment. Blood samples were centrifuged (1200×*g*, 5 min). Plasma supernatant was removed and stored at − 20 °C until used.

### Liquid scintillation counting

Plasma (10 μL), CSF and every injectate (1 μL of 1:10 dilution) were weighed and transferred into scintillation vials. In all experiments the radioactivity in the injectate was measured to confirm the uniformity of the injected material. Soluene350 (0.5 mL, PerkinElmer) was added to the brain samples and incubated overnight at 36 °C. Prior to adding scintillant two drops of glacial acetic acid (Sigma) were added to brain vials to neutralize the strongly alkaline Soluene350. All samples were mixed with 5 mL of scintillation fluid (Emulsifier-safe, PerkinElmer) and measured on the liquid scintillation counter (Tri-Carb 4910 TR, PerkinElmer). Counting was conducted in disintegrations per minute (DPM) for 5 min each with luminescence correction on. Vials containing control, non-radioactive tissues processed identically were also counted simultaneously to establish background counts (which were subtracted from all radioactive samples). Counts were normalized to the sample weight and expressed as DPM per µL or µg of sample. Results are described as concentration ratios, defined as a % of the counts (per µL or µg) in the compartment of interest (brain, CSF, maternal or fetal plasma) divided by the counts (per µL) in the plasma compartment of comparison (see also^2^).

### Transfer calculations

Placental transfer was calculated as:

$$Maternal \,to \,fetal \,placental\, transfer=\frac{{fetal \,plasma \,at \,time} \, x \,{(DPM/\upmu\text{ L})}}{{average\, maternal\, plasma\, up\, to \,time} \, x\, {(DPM/\upmu\text{L})}} \times \, 100\%$$

$$x=fetal \,plasma \,sampling\, time$$

Brain or CSF transfer was calculated as:

$$Brain \,or\, CSF\, transfer= \frac{Brain\, or \,CSF\, DPM/\upmu\text{L}}{Plasma\, DPM/\upmu\text{L}} \times 100\%.$$

### Ultraperformance liquid chromatography coupled with tandem mass spectrometry (UPLC-MS/MS)

Ten microliter plasma samples were spiked with an internal ^13^C-paracetamol standard (ring-^13^C_6_; Cambridge Isotope Laboratories Inc.; 10 μL of 10,000 μg/L in water) and diluted to 125μL in saline (105 μL). Proteins were precipitated by adding 375μL acetonitrile (ACN) before samples were mixed thoroughly and centrifuged (10,000×*g*) for 5 min. Supernatant was added to Captiva EMR lipid (Agilent) cartridges, centrifuged (30×*g*, 10 min; 450×*g*, 1 min) and flow-through was concentrated by vacuum evaporation to dryness (Thermo Scientific SpeedVac SPD131DDA; 42** °C**, 2 h). Dry powder was reconstituted with 2% methanol in water to 100 μL. UPLC-MS/MS was run on an Acquity H-class UPLC coupled with Vion Qtof mass spectrometer (Waters) using a Kinetex reverse phase C18 column (1.7 µm, 100 Å, 100 × 2.1 mm, Phenomenex). A gradient was established of 0.1% formic acid in water (A) and 0.1% formic acid in ACN (B), beginning with 5% B before being increased to 35% B at 3.5 min, 95% B at 4.5 min and maintained at 5% B at 5–7 min. Flow was at 0.3 mL/min and injection volumes 5 μL. The fragmentation product ions for each paracetamol metabolite are detailed in Table 1. Peak areas of extracted ion chromatograms of product ions were integrated through UNIFI software (Waters) corrected for background from measurements of blank plasma and compared to either the internal standard for paracetamol analysis or to external standard calibration curves prepared by spiking unlabelled compounds (Table 1) in control plasma samples and completing identical sample preparation for paracetamol-sulphate, paracetamol-glucuronide or paracetamol-glutathione metabolite analysis. The linearity of LC–MS/MS method for all metabolites in plasma measured here were tested at concentrations of 10 μg/L, 100 μg/L, 1 mg/L, 3.33 mg/L, 10 mg/L, 33.33 mg/L, 100 mg/L, 333.33 mg/L and 1 g/L (n = 6). The ranges of linear responses (R^2^ = 0.99) were 10 μg/L–33.33 mg/L (66 nM–220 μM) for paracetamol, 10 μg/L–33.33 mg/L (31 nM–102 μM) for paracetamol-glucuronide, 10 μg/L–1 g/L (22 nM–2.2 mM) for paracetamol-glutathione and 100 μg/L–333.33 mg/L (432 nM–1.4 mM) for paracetamol-sulphate. There are at least 6 calibrator levels for validating linear response range of each metabolite, which follows the current guidelines of US FDA Guidance for Industry, Bioanalytical Method Validation^14^. The carry-over in blank sample was also observed at less than 20% of peak area from metabolite at lower limit of quantification, after running each metabolite at upper limit of quantification. No further stringent validation of the LC–MS/MS method was done as it is intended for research use only, not clinical use.

**Table 1:** Compound and protocol information for paracetamol UPLC–MS/MS analysis.

Component name	Manufacturer	Code	Expected RT (min)	Expected m/z	Fragment m/z
^13^C_6_—Paracetamol	Cambridge Isotope Laboratories Inc	CLM-10619-PK	1.85	158	116
Paracetamol	SIGMA	103-90-2	1.85	152	110
					65
Paracetamol glucuronide	SIGMA	16110-10-4	1.36	328	110
					152
Paracetamol glutathione	NovaChem	A161223	1.92	457	382
					328
					152
Paracetamol sulphate	SIGMA	32113-41-0	2.31	232	152
					110

The compound being analysed (component name) and relevant manufacturer information is detailed along with column retention time (RT) and the mass to charge ratio (m/z) for the parent and fragment ions. The m/z transition: m/z 158 > 116, m/z 152 > 110, m/z 328 > 152, m/z 457 > 328 and m/z 232 > 152 were used for quantitative analysis of ^13^C_6_-paracetamol, paracetamol, paracetamol glucuronide, paracetamol glutathione, and paracetamol sulphate respectively, while other transitions were used as qualifiers.

#### Transcriptomic analysis: RNAseq

RNA extraction was completed using the RNeasy Plus Mini Kits and QIAshredder (Qiagen) for placenta using the RNeasy Plus Micro Kits (Qiagen) for fetal cortex, following manufacturers specifications. RNA quantity and purity were determined using a NanoDrop ND-1000 UV–Vis spectrophotometer (Thermo Scientific).

RNA samples were transported on dry ice to the Australian Genome Research Facility (AGRF) for Illumina, Next-generation sequencing. Runs were 100 bp single reads, providing raw FASTAq data. Data were processed using the Galaxy Australia platform and their online software packages^15^. Default parameters were used unless directly specified. Data were checked for read quality using fastQc read quality reports (Galaxy version 0.72). Alignment was conducted using HISAT2 (Galaxy version 2.1.0) using the reference genome for rat (rn6) and the reverse strand setting. For transcript quantification and differential expression analysis 3 different methods were employed. Expression was counted with feature counts (Galaxy version 1.6.4) using the reverse strand setting. The data were then fed through either DESeq2 (Galaxy version 2.11.40.6), Limma-voom (Galaxy version 3.38.3) or edgeR (Galaxy version 3.24.1) to receive differential expression analysis between treatment groups. Statistically different expression levels between relevant treatment groups were selected if present in at least 2 of the 3 datasets above. The statistical threshold of p < 0.05 for the adjusted P value of Limma-voom (Padj), DESeq2 (q value) or edgeR (FDR). This method of statistical selection minimizes the known false positives and false negatives that can be obtained due to the analysis pathway selected, ensuring all results can be found between multiple pipelines (see^16,17^). Gene synonym names were produced via annotatemyIDs (Galaxy version 3.7.0 + galaxy2).

### Autoradiography

Autoradiography was performed on brains of P4 and adult (6–10 week) rats, exposed acutely to paracetamol (15 mg/kg; 20 μCi adult, 2 μCi P4 of ^3^H-paracetamol) digoxin (30 μg/kg; 20 μCi adult, 2 μCi P4 of ^3^H-digoxin) or control animals not exposed to radioactivity. Brains were removed from terminally anaesthetised, exsanguinated animals; they were immediately snap frozen by placing on aluminium foil floating on liquid nitrogen; once frozen they were stored at − 80 °C.

Frozen brains were sectioned using a cryostat (Bright OTF5000) at − 20 °C chamber temperature. Sections were taken at 15 µm and placed on glass slides. Slides were apposed to ^3^H sensitive Biomax MS film with Intensifying screen (Kodak Biomax) and left to develop for 4 months. Activity range standards (0.07–6.5 nCi/mg tissue and 1–35 nCi/mg tissue; AMERSHAM) and control brains (no radiolabelled drug) were developed simultaneously. The images obtained were used to identify regional localisation of paracetamol in the brain.

### Statistics

RNAseq data analysis is detailed above, with significance set at p < 0.05. For all other experimentation statistical differences between treatment groups were determined by unpaired Student’s t-test using Prism 6.2 (Graphpad Software Inc) with significance set at p < 0.05.

## Results

### Developmental study: expression of ABC transporter and related enzyme at E19, P5 and in adults

ABC genes and their related metabolic enzymes identified in this study together with their estimated transcript numbers are shown in Supplementary Tables S1 and S2 respectively. Transcripts are described as present in a tissue if they had counts per million (CPM) of 1 or more. Transcripts of lower expression were considered not to have biological significance. Results are described in detail for eight ABC transporter transcripts that have evidence of efflux function in the tissues studied^18–21^. These are highlighted in Table 2 and were: *abcb1a, abcb1b, abcc1, abcc2, abcc3, abcc4, abcc5, abcg2*. Other ABC transporters in Supplementary Table S1 appear to have largely metabolic or intracellular transport functions, are not known to efflux drugs and primarily bind endogenous substrates such as lipids or peptides. Enzymes selected for analysis in this manuscript were in the subfamilies of cytochrome P450 (CYP), glutathione-s-transferase (GST), sulfotransferase (SULT) and uridine 5′-diphospho-glucuronosyltransferase (UGT). There is insufficient information available to establish which enzymes have functional links with the ABC efflux transporters identified in each tissue/age.

**Table 2:** Expression of key ABC transporters associated with drug efflux at placental and blood–brain barriers in control tissue.

Gene	Placenta	Brain				Choroid plexus			
	E19	E19	P5	Adult (male)	Adult (female)	E19	P5	Adult (male)	Adult (female)
*Abcb1a*	79	8	12	51	50	2	2	2	1
*Abcb1b*	211	4	4	0.8	0.6	2	1	0.4	0.6
*Abcc1*	48	22	23	7	7	32	28	88	102
*Abcc2*	15	3	4	5	5	0.7	0.6	2	2
*Abcc3*	12	1	1	1	2	9	10	2	2
*Abcc4*	46	10	15	13	12	19	24	90	115
*Abcc5*	32	90	62	107	116	77	60	52	45
*Abcg2*	25	2	6	9	22	3	3	0.6	1

Values are average counts per million (CPM; n = 4) measured by RNAseq and analysed using EdgeR.

Results are divided into sections that describe: (i) the expression of transporters and enzymes in placenta, fetal brain and choroid plexus at E19, (ii) analysis of transcriptomes for brain and choroid plexus of neonatal pups and (iii) datasets for adult brain and choroid plexus. Adult results are presented for male and female cohorts in order to identify counts that relate to sexually mature females. Subsequent sections deal with gene expression estimates following treatment of animals with paracetamol at different ages and different treatment regimens. Finally, results of permeability experiments to test functional responses are described.

### E19 placenta, brain and choroid plexus

#### Placenta

In control placentas there were 36 ABC transporter genes identified (Supplementary Table S1). Of these *abcf1, abcg1* and *abcb1b* had CPM values over 200. All 8 ABC transporters with known efflux functionality were identified with PGP transcripts (*abcb1a, abcb1b)* having the highest expression (Table 2)*.*

There were 51 metabolising enzyme genes associated with ABC-mediated transport present in the E19 placenta: 27 CYPs, 18 GSTs, and 4 SULTs. Although only 2 UGTs were identified in the tissue, *uggt1* had the second highest expression for all metabolizing enzymes investigated (Supplementary Table S2). Most of these enzyme genes have not previously been identified in placenta (cf.^22,23^).

#### Brain

**Figure 1:**
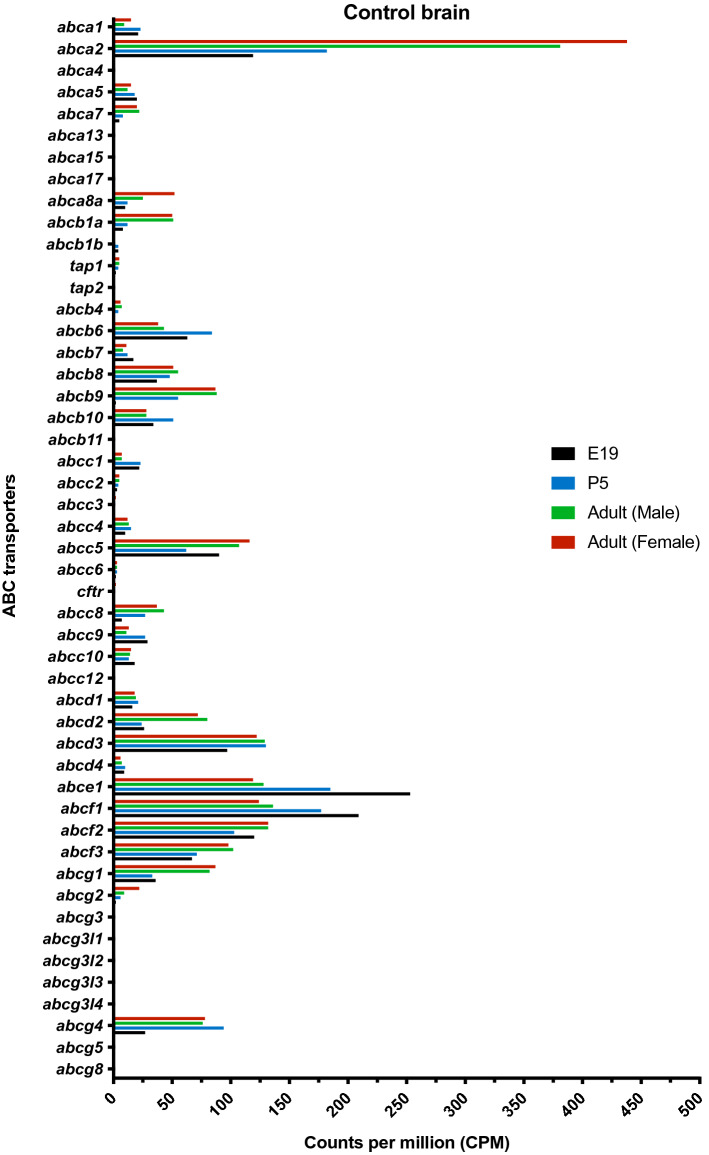
Expression of ABC transporters identified in rat brain at different ages. Average, normalized counts per million (CPM) for ABC transporters in brain at different ages (n = 4) as measured by RNAseq using EdgeR analysis. Ages indicated by colours: black E19, blue P5, green male adult, red female adult. In general expression levels appear to be similar at all ages or higher in the adult. But a few were higher in the fetus (*abce1*, *abcf1, abcc9, abcc1*).

In E19 brain 33 ABC gene transcripts were identified (Supplementary Table S1). Of these *abce1 and abcf1* were highly expressed (> 200 CPM) while *abcc5* and *abcc1* were expressed above 10 CPM (Table 2). Figure 1 summarises ABC transporters identified in brain at different ages studied, together with their expression levels. Thirty-six enzymes associated with ABC genes were detected (Supplementary Table S2). Of these *sult4a1* (a sulphotransferase) and *gstm7* (a glutathione transferase) were the only ones expressed above 10 CPM that were not also expressed at this level in placenta. Enzymes *uggt1 and cypor* were expressed above 100 CPM in both placenta and fetal brain at E19.

#### Choroid plexus

In E19 choroid plexus 39 ABC genes were identified with *abcd3, abca2, abce1 and abcf1* highly expressed (> 100 CPM) while *abcc5, abcc1 and abcc4* were expressed above 10 CPM (Table 2). Forty-seven ABC associated enzymes were identified in E19 choroid plexus. GSTs had high expression, including *gstm1* (446 CPM) and *gstm2* (200 CPM).

### P5 brain and choroid plexus

#### Brain

In the P5 brain 35 ABC efflux transporters were identified (Supplementary Table S1) with *abce1, abca2 and abcf1* having highest expression. All 8 ABC transporter transcripts with known efflux function (Table 2) were identified in the tissue, with *abcc5* (0.7-fold) and *abcb1a* (1.6-fold) the only transcripts with significant differences to E19. There were 39 enzymes identified: 17 CYP enzymes, 17 GSTs, 3 SULTs and 2 UGTs with 22 enzymes having significantly (p < 0.05) higher levels at P5 than at E19, including *cyp46a1* that increased 13-fold to over 100 CPM.

#### Choroid plexus

In choroid plexus there were 40 ABC efflux transporters identified (Supplementary Table S1). Only 4 ABC efflux transporters had CPM above 3: *abcc5, abcc1, abcc4, abcc3* (Table 2)*.* In P5 choroid plexus 45 enzymes were identified, 36 of which were also present in the P5 brain. Of these *gsta1* had the second highest expression (to *gstm1*) and may represent a choroid plexus specific enzyme that was not identified in P5 brain. In contrast, *sult4a1* had over 30 times higher expression in P5 brain compared to choroid plexus. Overall compared to the brain, 30 enzymes had significantly higher transcript numbers in the P5 choroid plexus, whereas there were 16 with significantly lower transcript numbers (Supplementary Table S2). Expression of 9 ABC transporter genes was significantly higher at P5 compared to E19, including *abca4* (18-fold), and 7 were significantly higher at E19, including *abcd2* (15-fold) and *abcc5* (1.3-fold). Sixteen ABC associated enzymes were significantly higher in P5 than E19 choroid plexus, including *gsta1* (threefold) and *sult1a1* (threefold). Eleven enzymes were significantly higher at E19, including *cyp26b1, gstm7* and *uggt1* (all twofold).

### Adult brain and choroid plexus

#### Sex differences

In the brain of both males and females 35 ABC transporters were identified, with *abcc5, abcb1a, abcg2* and *abcc4* expressed above 10 CPM (Table 2). The only transporter that had significantly different expression between the sexes was *abcg2* (2.3-fold higher in females*)*. Fifty-five enzymes were present in both the male and female adult brain. Of these *gstm4* (1.4-fold higher in males) and *sulft5a1* (2.8-fold higher in females) were the only significant differences between sexes.

Thirty-seven ABC transporters were identified in the choroid plexus of both males and females, with *abcc4, abcc1* and *abcc5* expressed above 45 CPM (Table 2). The only transporter that had significantly different expression between the sexes was *abcf3* (1.1-fold higher in females). Forty-five enzymes were present in both male and female adult choroid plexus. *Cyp39a1* (1.9-fold higher in males) was the only significant differences between sexes. Overall, there were minimal differences in ABC efflux transporters and enzymes in the brain and choroid plexus of male and female adult rats.

#### Brain

Of the ABC transporters identified in the P5 or adult brain 16 were higher in adults (including *abcc7,* fivefold; *abcb1a,* fourfold; *abcg2* and *abcc5* twofold) and 9 were significantly higher at P5 (including *abcb1b,* sevenfold; *abcc1,* threefold). Of the enzymes identified in the P5 or adult brain 42 were higher in adults (including *cyp11b2,* 502-fold; *gsta1,* 106-fold; *ugt8,* 11-fold) and 6 enzymes had higher expression at P5 (including *cyp1b1,* ninefold; *uggt1,* twofold).

#### Choroid plexus

Of the ABC transporters identified in the P5 or adult choroid plexus 21 were significantly higher in adults (including *abca4,* eightfold; *abcc4,* fourfold; *abcc1,* threefold) and 13 were significantly higher at P5 (including *abcc9,* sevenfold; *abcg5,* fivefold; *abcc3,* fourfold). Of the enzymes identified in the P5 or adult choroid plexus 19 were significantly higher in adults (including *cyp27a1,* fivefold; *ugt8* and *gsta4,* threefold) and 13 were significantly higher at P5 (including *sult4a1,* fivefold and *gstm1, gstm2, sult1a1,* threefold).

Thirty-three ABC transporters were significantly different between adult brain and choroid plexus for both male and female cohorts and 19 were significantly higher in choroid plexus (including *abca4* 550-fold; *abcc1,* 12-fold; *abcc4* sevenfold) while 14 were significantly higher in brain (including *abcd2,* 185-fold; *abcb1a,* 34-fold; *abcg2,* 18-fold; *abcc5,* twofold). Twenty-four ABC transporter associated enzymes were significantly higher in the choroid plexus (including *cyp4f17, mgst, cyp4f6* > tenfold) and 16 were significantly higher in brain (including *sult4a1,* 276-fold; *ugt8,* 50-fold; *mgst3,* 31-fold).

### Concentration of paracetamol and its metabolites in fetal and maternal plasma

The concentration of paracetamol and its metabolites in maternal and fetal plasma following acute and chronic maternal administration of the clinical (15 mg/kg) dose is listed in Table 3. Maternal plasma concentration of un-metabolized paracetamol 40 min after the final injection was 6.4 mg/L (42 μM) in the acutely treated dam and 6.0 mg/L (40 μM) in the chronically treated dam. In fetal (E19) plasma between 30 and 120 min post-injection concentration of paracetamol was approximately 3.0 mg/L (20 μM; 3.3 mg/L ± 0.7 acute, 2.7 mg/L ± 0.7 chronic). Estimated placental transfer of paracetamol was approximately 50% for both treatment lengths (51% ± 10 acute, 46% ± 10 chronic). Paracetamol-glutathione was present in the maternal plasma at extremely low levels (72–88 nM; 33 μg/L acute, 40 μg/L chronic) and was generally below the limit of detection (10 μg/L) in fetuses. However, paracetamol-sulphate and paracetamol-glucuronide were present in plasma at much higher levels in the dam than the un-metabolised form. These metabolites also had very different maternal and fetal plasma profiles for acute and chronic treatment groups. Paracetamol-glucuronide was present in the maternal plasma of the chronically treated dam (101 μM; 33.0 mg/L) at almost twice the concentration of the acutely treated dam (53 μM; 17.3 mg/L). In contrast, paracetamol-glucuronide concentration in the E19 plasma of the chronic group was significantly less (6 μM; 1.9 mg/L ± 1.1; 0.4-fold, p < 0.05) than in the acute group (14 μM; 4.6 mg/L ± 0.8). These results corresponded to an estimated placental transfer of 6% ± 3 in the chronic group, which was significantly lower (0.2-fold, p < 0.01) than the acute group (27% ± 5). Like paracetamol-glucuronide, chronically treated dams had a higher plasma concentration of paracetamol-sulphate (208 μM; 48.1 mg/L) than the acutely treated dam (171 μM; 39.5 mg/L). The concentration of paracetamol-sulphate was significantly lower (0.4-fold, p < 0.05) in E19 pups exposed chronically to maternal paracetamol (17 μM; 4.0 mg/L ± 1.4) compared to those exposed acutely (40 μM; 9.2 mg/L ± 2.4). Estimated placental transfer of paracetamol-sulphate was significantly lower (0.3-fold, p < 0.05) for the chronic group (8% ± 3) compared to the acute group (23% ± 6).

**Table 3:** The concentration of paracetamol and its metabolites in plasma after acute (single dose; E19) or chronic (5 day twice daily; E15–E19) maternal injection of 15 mg/kg paracetamol.

	Paracetamol		Paracetamol-glutathione		Paracetamol-glucuronide		Paracetamol-sulphate	
	Acute	Chronic	Acute	Chronic	Acute	Chronic	Acute	Chronic
Maternal plasma	42 μM	40 μM	72 nM	88 nM	53 μM	101 μM	171 μM	208 μM
Fetal plasma	22 μM ± 4	18 μM ± 5	N.D.	N.D.	14 μM ± 2	6 μM ± 4	40 μM ± 11	17 μM ± 6

**Figure 2:**
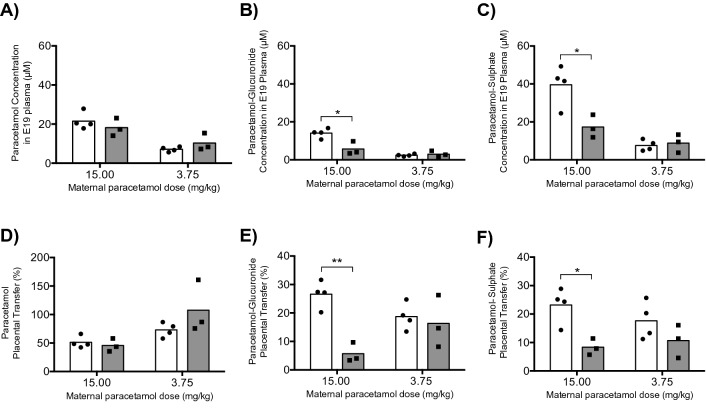
Levels of paracetamol and its metabolites in fetal and dam plasma. The concentration of paracetamol, paracetamol-glucuronide and paracetamol-sulphate in E19 plasma are shown in (**A–C**) respectively following 3.75 or 15 mg/kg acute (open bars) or chronic (grey bars) regimens. The placental transfer (%) between maternal and fetal circulation is shown for the three paracetamol structures in (**D–F**). Measurements were made using UPLC-MS/MS (see “Methods” section). Values from individual experiments are shown; n = 3–4. *p < 0.05; **p < 0.01.

Fetal results are ± S.D. For animal numbers see Fig. 2.

*N.D.* not detected.

The concentrations of paracetamol and its metabolites in maternal and fetal plasma following acute and chronic maternal administration of a reduced (3.75 mg/kg) dose are listed in Table 4. The concentrations of paracetamol and all metabolites in maternal and fetal plasma were lower for the 3.75 mg/kg (Table 4) than 15 mg/kg dose (Table 3). In contrast to the results of the 15 mg/kg regimen, the 3.75 mg/kg regimen had no significant differences between acute and chronic placental transfer of paracetamol metabolites (Fig. 2).

**Table 4:** The concentration of paracetamol and its metabolites in plasma after acute (single dose; E19) or chronic (5 day twice daily; E15–E19) maternal injection of 3.75 mg/kg paracetamol.

	Paracetamol		Paracetamol-glutathione		Paracetamol-glucuronide		Paracetamol-sulphate	
	Acute	Chronic	Acute	Chronic	Acute	Chronic	Acute	Chronic
Maternal plasma	10 μM	10 μM	24 nM	N.D.	13 μM	18 μM	43 μM	83 μM
Fetal plasma	7 μM ± 1	11 μM ± 4	N.D.	N.D.	2 μM ± 1	3 μM ± 2	8 μM ± 3	9 μM ± 5

Fetal results are ± S.D, for animal numbers see Fig. 2.

*N.D.* not detected.

### Placental permeability to paracetamol at E19

**Figure 3:**
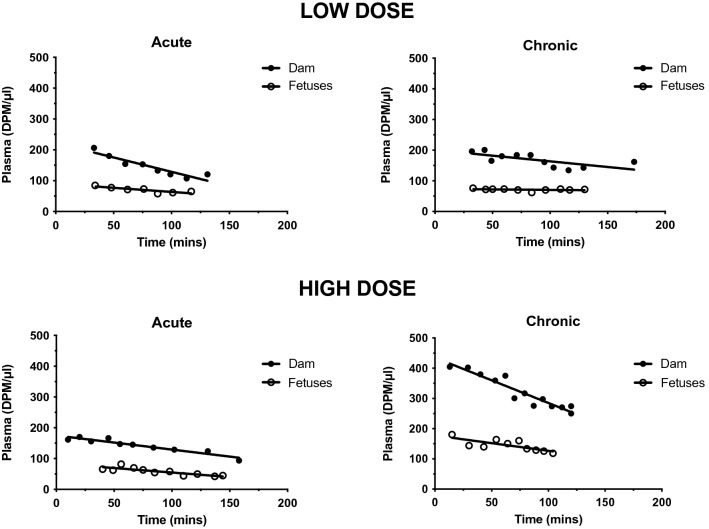
Activity levels (DPM/μl) of ^3^H-paracetamol (acute and chronic experiments) in E19 fetal plasma. Above: low dose, 3.75 mg/kg. Below: high dose, 15 mg/kg. The maternal plasma levels for both acute and chronic experiments declined progressively throughout the experimental period. The fetal plasma levels also declined during this period, except in chronic low dose experiments. The levels in maternal and fetal plasma were higher with chronic high dose treatment. The lower levels for each drug in fetal plasma indicates a substantial restriction of drug transfer across the placenta. Fetal plasma, open circles. Maternal plasma, filled squares. Lines fitted by Least Squares Linear Regression, curve fitted by Least Squares Exponential Decay (one phase). Data for high dose experiments from Ref.^2^.

**Figure 4:**
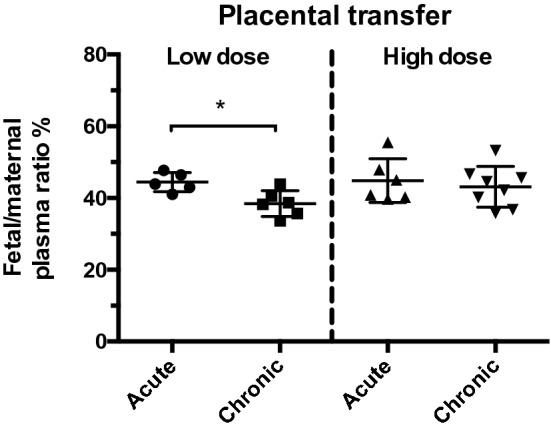
Placental transfer of ^3^H-paracetamol at E19. Fetal/maternal plasma concentration ratio (%) for low and high dose paracetamol labelled with ^3^H following acute or chronic treatment. The low dose chronic treatment group had a ratio (38%) that was significantly below the acute low dose group. Overall the other ratios were about 45%. Symbols are plasma samples from individual pups. Mean ± SD; n = 5–8. *p < 0.05. High dose data from (Koehn et al.^2^).

Figure 3 illustrates the level and time course of ^3^H-paracetamol transfer from maternal blood to fetal blood at E19 in animals treated with low doses of paracetamol either acutely or chronically (see above). Results of similar experiments with the high doses are shown below (from Ref.^2^). The levels of radiolabelled drug were similar in the low and high doses of acutely and chronically treated animals but higher (about twofold) in the high dose chronically treated animals. The plasma levels of radioactivity declined in both fetuses and mothers during the course of the experiment but more so in the mothers’ plasma. Figure 4 shows estimates of placental transfer of radiolabelled paracetamol (fetal/maternal plasma ratios %) for the four treatment conditions. Overall fetal/maternal plasma ratios were about 45% indicating a degree of placental protection against entry of paracetamol from the mother into the fetus. This was similar to the ratios for acute and chronic high dose paracetamol when measured by UPLC-MS/MS (see above).

### Brain barrier permeability to paracetamol at E19

**Figure 5:**
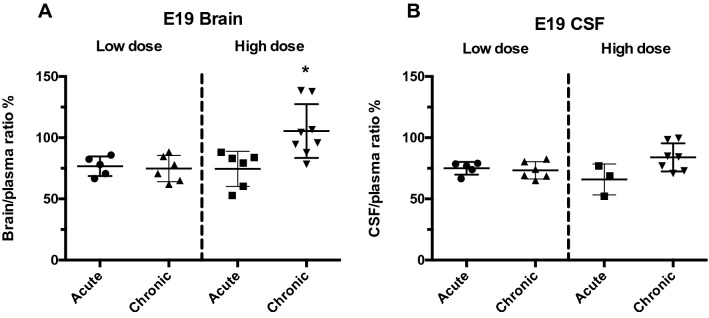
Entry of ^3^H-paracetamol into fetal brain and CSF at E19 following low and high doses administered acutely or chronically. Results are expressed as brain/plasma and CSF/plasma ratios (%). The ratios were similar for brain and CSF. The chronic high dose brain mean was significantly higher than the other treatment groups. Horizontal bars are mean ± SD. Symbols are results from individual animals, *p < 0.05 for difference between high dose and all other treatments. High dose data from^2^.

Entry of ^3^H-paracetamol into brain and CSF at E19 following acute or chronic administration of paracetamol at low or high doses is shown in Fig. 5 expressed as brain/plasma and CSF/plasma ratios (%). Brain to plasma ratio in the chronically treated high dose group was significantly higher than for acute treatment (p < 0.05). Chronic treatment with the low dose of paracetamol did not change the brain/plasma ratio compared to acute treatment. No significant changes in the CSF/plasma ratio were seen with either dose or treatment regime (Fig. 5).

### Autoradiography

**Figure 6:**
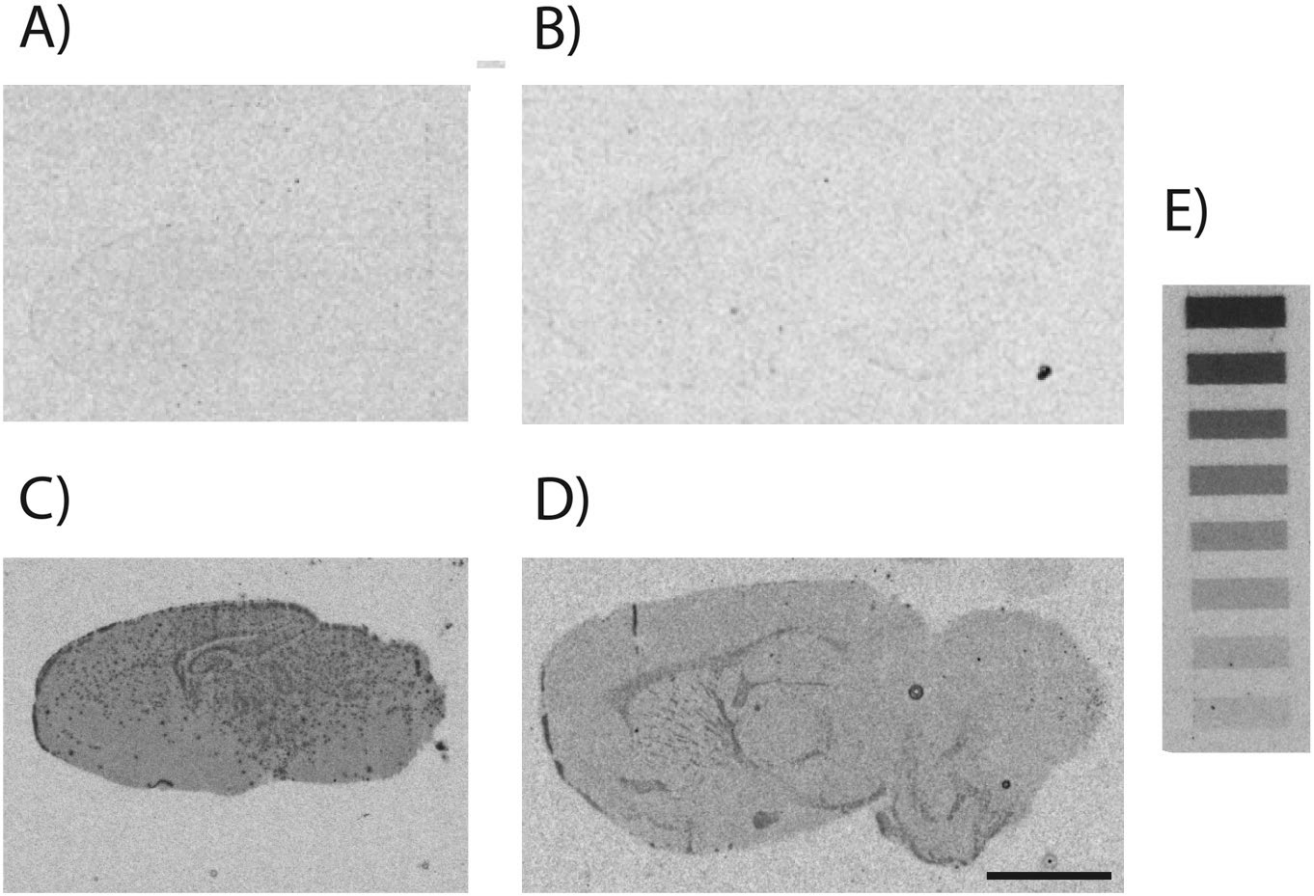
Autoradiographic images of rat brains. (**A**) Control P4, no radioactivity exposure. (**B**) adult exposed acutely to 30 μg/kg digoxin with 20 μCi ^3^H-digoxin (negative control) (**C**) P4 exposed acutely to 15 mg/kg paracetamol with 2 μCi ^3^H-paracetamol or (**D**) adult exposed acutely to 15 mg/kg paracetamol with 20 μCi ^3^H-paracetamol. (**A**) Control and (**B**) digoxin brains show only background levels of radioactivity. In the adult brain (**D**) there was widespread distribution of paracetamol with accumulation in the corpus callosum and striatum white matter. The intensity of the image of the P4 brain (**C**) indicates widespread distribution and greater entry of paracetamol compared to adults (all films were exposed for the same length of time). Adult control showed only background exposure (not illustrated). Scale bar (5 mm) in bottom right applies to all brain sections. (**E**) Radioactive ^3^H standards (Amersham, 3.1–35 ng/mg) were exposed on the same film as the brain sections.

Autoradiography of ^3^H-labelled paracetamol was used to determine its distribution in the brain following acute i.p. injection. This is illustrated in Fig. 6 in sagittal sections of brains from P4 (Fig. 6C) and adult (Fig. 6D) rats exposed in vivo to ^3^H-paracetamol (15 mg/kg) acutely. Figure 6A,B shows the minimal background level in control brains that were either: (i) not exposed to any radiolabelled drug (Fig. 6A) and (ii) a brain that was exposed to radiolabelled digoxin (Fig. 6B), which only enters the adult brain to a limited extent^2^. When compared to radioactive standards on the same film, the level of ^3^H-radioactivity in both Fig. 6A and Fig. 6B were below the lowest detection of 3.1 nCi/mg. Sections from brains exposed to paracetamol show its uniform distribution in the P4 brain, with granular deposits throughout (Fig. 6C). Quantification from ^3^H standards on the film indicates that radioactivity in the P4 brain was approximately 18.4 nCi/mg. Figure 6D shows paracetamol in a similar uniform distribution across the adult brain, with darker deposits in the corpus callosum and the striatum. Quantification in relation to ^3^H standards on the film indicates that radioactivity in the adult brain was approximately 5.1 nCi/mg. This confirms the earlier observation that much less paracetamol enters the adult brain than the developing brain^2^ see “Discussion” section.

### Effect of paracetamol exposure on expression of ABC transporters and related enzyme at E19 in placenta, fetal brain and adult brain

#### E19 placenta following paracetamol treatment of their mothers (acute and chronic)

Compared to untreated (control) placentas, both acute and chronic treatment decreased placental expression of several ABC transporters: *abcc2* (16-fold acute, 38-fold chronic), *abcc8* (11-fold acute, 49-fold chronic) and *abcc6* (threefold acute, fivefold chronic). Chronic treatment also significantly decreased *abcg2* (twofold). No transporters increased expression in the placenta after acute or chronic treatment.

Enzyme *cyp2c22* decreased from 7 CPM in control placentas to not present (< 1 CPM) in those treated chronically. Several additional CYP and GST enzymes were significantly different after acute or chronic treatment, however counts (< 4 CPM) or fold changes (< 1.6-fold) were low (see Supplementary Table S2).

#### E19 fetal brain and choroid plexus following paracetamol treatment of their mothers (acute and chronic)

In E19 brain there was an increase in *abcb9* (twofold) with acute treatment and *abcc5* (1.3-fold) with acute and chronic treatment. Enzymes *cyp26b1* (threefold) and *cyp46a1* (1.5-fold) increased with acute treatment and *gstm2* (threefold) decreased with chronic treatment. *sult1a1* decreased from 2 CPM in control brains to < 1 CPM after acute or chronic treatment. Chronic treatment significantly decreased some transporters: *abcd2* (sixfold), *abcb1b* (twofold) and enzymes *cyp4f4* (threefold), *sult4a1* (twofold) in the E19 choroid plexus. Nine enzymes significantly increased expression, including *cyp27a1, cyp2t1, sult1a1* (all twofold) and *gstm1* (1.3-fold, 575 CPM).

#### P5 brain and choroid plexus, acute and chronic paracetamol treatment

After chronic paracetamol treatment at P5 few significant differences in ABC transporters or enzymes in the brain or choroid plexus were observed. No significant differences exceeded 1.8-fold (See Supplementary Tables S1, S2).

#### Adult brain and choroid plexus, acute and chronic paracetamol treatment

Several transporters increased expression in adult brains after acute (9 transcripts) or chronic (6 transcripts) treatment, including *abcc1* (twofold), *abca5* (1.9–twofold), *abcc9* (1.8-fold), *abcb7* (1.6–1.8-fold) and *abcb1b* (1.4–1.6-fold) in both groups. No ABC transporters decreased expression in the adult brain after either treatment length. After both acute and chronic treatment the same 3 enzymes increased expression: *cyp1b1* (fourfold), *uggt1* (twofold) and *gstm2* (twofold). There were 6 GST (*gstk1, gstm1, gstm7, gsto1, gstp1, gsto2*) and two CYP (*cyp4f1, cyp2t1*) that were significantly decreased in the brain of both acute and chronically treated adults (1.3–twofold). In adult choroid plexus expression of 17 ABC transporters decreased with chronic treatment, including *abca1, abcc1 and abcc9* (all twofold). Only two transporters, *abcd3* and *abce1* significantly increased expression following chronic treatment, however fold changes were small (1.1–1.2-fold). Chronic treatment significantly increased 6 enzymes including *gsto2* and *uggt1* (both twofold). The 6 enzymes that were significantly decreased in the adult choroid plexus were all GSTs (*gsta4, mgst1, gsta6, gstcd, gstk1, gstz1*) with low fold changes (< 1.7 fold; Supplementary Table S2).

## Discussion

In the present study the transcriptome of the E19 placenta as well as the brain and choroid plexus from three developmental ages: E19 (fetal), P5 (postnatal) and adult (female and male) have been analysed with respect to expression of ABC efflux transporters and related enzymes. Changes to transcript expression after acute or chronic paracetamol treatment was assessed and the levels of paracetamol and its metabolites measured in E19 pregnant dams.

Transfer of paracetamol across the placental and blood–brain barriers differed depending on dose and treatment length (Figs. 4, 5). Efflux capacity of the barriers in question and concentration of paracetamol and its metabolites that may bind to efflux mechanisms on those barriers were investigated to explain differences in observed transfer levels of the drug.

UPLC-MS/MS analysis allowed for quantification of paracetamol and its metabolites in the blood of pregnant rats. The results indicate that a 15 mg/kg dose produced the maternal plasma concentration of approximately 6 mg/L. This is similar to the value of 6.5 mg/L reported previously^24^ for the same dose. Reviews of the literature suggest that effective plasma paracetamol concentrations in people are between 5 and 20 mg/L, but most studies were not in pregnant patients^25^. UPLC-MS/MS studies in humans have shown that a 1 g oral dose (approximately 15 mg/kg in a 70 kg human) results in a plasma concentration at around 10 mg/L 30 min after ingestion^26,27^. The plasma concentrations in our study were similar. Previous studies in rats and humans have found that the major metabolites of paracetamol are paracetamol-sulphate^28,29^ and paracetamol-glucuronide^24,26,30^. Thirty minutes after administration maternal plasma paracetamol-sulphate was twice the concentration of paracetamol-glucuronide, which was threefold higher than un-metabolised paracetamol (Tables 2, 3). Paracetamol-glutathione was present in blood at low concentrations, as reported previously^30^.

Transfer of ^3^H-paracetamol across the E19 placenta was 40–50% for both doses (3.75 and15mg/kg) and both treatment lengths (acute and chronic; Fig. 4) with the exception of placental transfer in chronically treated dams at the lower dose, where the fetal/maternal ratio was slightly but significantly lower (p < 0.05) than for the acute dose (Fig. 4). UPLC-MS/MS metabolite concentration measurements in maternal and fetal plasma only provide “estimates” of placental transfer because metabolites could be produced by either the mother or the fetus. However, paracetamol-glucuronide and paracetamol-sulphate had lower levels of placental transfer (< 25%) compared to the parent molecule (> 50%; Fig. 2). In livers from mice in which various ABC transporters had been knocked out there was evidence of involvement of Abcg2, Abcc3 and Abcc4 in excretion of sulphate and glucuronide metabolites of paracetamol^31,32^. Similar mechanisms may account for restricted transfer of these metabolites at the placental barrier. The placental transfer of paracetamol from liquid scintillation counting (40–50%; Fig. 4) was similar to UPLC-MS/MS measurements (50–100%, Fig. 2). Previous clinical studies found that concentrations of paracetamol in maternal and fetal blood were similar (around 100%), which was interpreted as reflecting blood flow limited diffusion of paracetamol across the placenta^33^. The lower ratio in our experiments implies a degree of restriction. In a study of perfused human placentas in ex vivo preparation it was found that the fetal/maternal ratio was 45%^34^ as in our experiments. It may be that the difference is that in the clinical studies samples of cord and maternal blood were obtained at the time of labour when the separation of the placenta may affect its permeability.

Radiolabelled paracetamol transfer from fetal blood into fetal brain and CSF was approximately 75% for acute and chronic 3.75 mg/kg maternal treatment (Fig. 5). UPLC-MS/MS data indicate that the concentrations of paracetamol, paracetamol-glucuronide and paracetamol-sulphate in fetal plasma were similar for both treatment lengths (Tables 2, 3; Fig. 2). This indicates that 3.75 mg/kg maternal treatment will expose the fetus to similar concentrations of paracetamol metabolites and result in similar transfer of combined paracetamol compounds into the brain in acute and chronic regimens. In contrast, radiolabelled paracetamol transfer from E19 fetal blood into brain was significantly higher (p < 0.05) for chronic 15 mg/kg treatment (100%) than acute treatment (75%) see Fig. 5. UPLC-MS/MS analysis identified that chronic 15 mg/kg pups had significantly lower concentrations of paracetamol metabolites in plasma than after acute treatment but similar concentrations of parent paracetamol (Table 3, Fig. 2).

Radiolabelled paracetamol transfer from adult blood to brain (15 mg/kg) has been previously reported to be lower after chronic exposure compared to acute treatment^2^. In maternal plasma there were higher levels of paracetamol-glucuronide and paracetamol-sulphate after chronic treatment (Tables 3, 4). Unlike fetal blood, the levels of metabolites in adult circulation with chronic treatment were higher, which may explain the decrease in overall paracetamol transfer from blood to brain with chronic treatment^2^.

Autoradiography of radiolabelled paracetamol provided spatial evidence that paracetamol distributes widely throughout the brain at neonatal and adult ages. In the adult brain (Fig. 6) darker exposure can be observed in the myelin rich corpus callosum and white matter bundles within the striatum^35^. Paracetamol is a lipid soluble compound and therefore it is likely to localise in lipid components of the brain such as myelin. Other highly lipid soluble drugs, such as ^14^C-Carbaryl, have also shown corpus callosum localisation in autoradiography images^36^. A comprehensive study of a large number of psychoactive drugs used autoradiography to quantify the distribution of the drugs in different brain regions including white matter^37^. At P4 the brain contained noticeable granular deposits of paracetamol spotted throughout the tissue as well as highlighted staining in the hippocampal region (Fig. 6). It is possible that these areas of ^3^H-paracetamol indicate localisation within some cerebral blood vessels or affinity for certain cell types within the brain. Additional studies would be required to explain this pattern of localisation.

The E19 placenta had high expression (> 10 CPM) of ABC transporters known to export drugs and metabolites (Table 2). The transcripts for PGP (*abcb1a; abcb1b*) had the highest expression, followed by *abcc1, abcc4, abcc5, abcg2, abcc2* and *abcc3*. Previous publications have localized PGP, BCRP (*abcg2*) and MRP (*abcc* subfamily) transporters to the maternal facing barrier of placental syncytiotrophoblast cells in several species, where they decrease molecular transfer from mother to fetus (for review see^38^, for human data see^39–42^: and rat^43^. RNAseq data identified high levels of numerous GST and CYP enzymes in the E19 placenta (Supplementary Table S2). Apart from *uggt1*, which largely interacts with intra-organelle glycoproteins^44^, the level of SULT (4 transcripts; < 6CPM) and UGT (1 transcript, 2 CPM) enzymes were low in the E19 placenta.

Acute paracetamol treatment significantly altered ABC transporter and enzyme expression in the placenta, brain and choroid plexus (Supplementary Table S3A–C). This indicates that even after a 30-min circulation period paracetamol can elicit changes to transcript expression. It is not likely that these changes would translate to functional differences in paracetamol transport during the early stages of the first dose, however it does suggest that any differences in transfer observed at 5 days of chronic treatment in this study may also occur at earlier times.

The expression of several ABC transporters (*abcc2, abcg2*) and enzymes (*cyp2c22*) decreased in the placenta after chronic maternal paracetamol treatment compared to untreated controls. There were no ABC efflux related products that increased. This may indicate that changes observed in the transfer of paracetamol metabolites with chronic treatment (Tables 3, 4) were not due to ABC-related gene up-regulation in the placenta but rather that these changes were due to alterations in metabolite production in the maternal and fetal compartments or changes to paracetamol transport mechanisms that are yet to be discovered.

Over the period of development studied there were changes in the expression of ABC transporters and related enzymes in the brain and choroid plexus. The two major transporters in the adult blood–brain barrier, PGP and BCRP, have been previously identified from early fetal periods^45^ and the transcript levels increase during development^46^. The results in the current study support these findings (Table 2). *abcc1, abcc9*, *abcb6* and *abce1* transporters decreased over the course of development, and a large number of ABC transporters and associated enzymes increased. The enzymes included GST (e.g. *gsta1*), UGT (e.g. *ugt8*), SULT (e.g. *sult1d1*) and CYP (e.g. *cyp46a*) enzymes (see Supplementary Table S3A–C). The relative contribution of changes to either enzymatic or transporter expression to developmental differences in drug efflux at the blood brain barrier^2^ is an interesting area of future research.

Similar to the brain, the two major transporters in the adult blood-CSF barrier, MRP1 (*abcc1*) and MRP4 (*abcc4*), have been identified from early fetal periods^45^ and increase in transcript expression during development^46^. The choroid plexus results in the current study also support these findings (Table 2). Changes to transporters over the course of development such as decreases to abcc3, warrant further examination (see also Supplementary Table S1). Over the course of development many enzymes both increased and decreased expression (Supplementary Table S2). This included an increase of *gsta1* and *gsta4* and a decrease of *gstz1* and *gstm2*. Changes to the expression of certain enzymes combined with the overall increase in expression of major drug efflux transporters at blood–brain and blood-CSF barriers may explain differences in molecular permeability into the central nervous system observed over development for a range of molecular classes^2^.

Entry of paracetamol into the adult brain decreases over the course of chronic treatment^2^. RNAseq analysis showed that after chronic treatment there was a significant increase in the level of ABC transporters, such as *abcc1* (twofold), and enzymes such as *gstm2* (twofold). Previous single cell RNAseq studies have identified *abcc1* and *gstm2* in vascular endothelial cells, however they are also present in many other cell types in the brain^47^. MRP1, the protein product of *abcc1*, has been localized to luminal membranes of brain endothelial cells^45,48^, however these results are contradicted by other studies^49^. Following chronic maternal treatment of clinical doses of paracetamol there was increased drug transfer to the E19 brain, the opposite of what was identified in the adult brain^2^. RNAseq analysis showed that enzymes *sult1a1* (fourfold) and *gstm2* (threefold) were expressed less in the E19 brain after chronic maternal exposure. The regulation of *gstm2* in adult and E19 brains correlates with changes of paracetamol entry to the brain at both ages with chronic treatment^2^. However, the low levels of paracetamol-glutathione in fetal blood (Tables 3, 4) suggests that changes in glutathionation at brain barriers by *gstm2* may not be sufficient to explain the differences in their permeability following chronic treatment. Specific *abcc1* regulation in adult but not fetal brains may explain a broader range of metabolite restriction and represent an age-specific alteration to barrier efflux.

### Study limitations and future directions

Although UPLC-MS/MS analysis provided valuable insight into the transfer of paracetamol metabolites from maternal to fetal blood, metabolite analysis was not complete for those with very low concentrations in fetal and adult brain. This would require substantial optimization for adequate extraction of each metabolite from the brain and to obtain comparable extractions from brains at different ages. Such a study might highlight specific changes in different drug metabolites reaching the brain at different developmental stages. However, transfer measurements of ^3^H-paracetamol into fetal brain was conducted with liquid scintillation counting, which had the advantage of not requiring extraction from brain tissue to measure. These values do have the limitation of not providing complete identification of what paracetamol forms are being measured in each tissue; but that information has been provided for plasma from the UPLC-MS/MS measurements; therefore the two methods should be treated as complimentary.

The regulation of some ABC transporters and enzymes with chronic treatment of high dose paracetamol, such as *gstm2*, has not been investigated at lower doses. Dose response analysis of some transcripts identified in this study, alongside similar studies investigating metabolite concentrations at different doses, may provide evidence of specific drug concentrations that could be administered to result in stable drug distribution over extended treatment periods. It should also be noted that although ABC transporters present a major family of efflux exporters at barrier interfaces, other transport systems do exist. Extensive analysis of lesser known blood–brain and placental transport systems may discover transporters that interact with paracetamol or its metabolites that have not yet been identified. Future investigation of protein levels to identify the influence of translation, protein trafficking and utilization on overall transporter levels and function would complement the transcriptomic analysis of the present study.

The rat model used in this study allowed for large-scale, exploratory analysis of blood–brain and placental barriers and their effectiveness under different drug treatment regimens. Rats have several similarities to humans in terms of ABC transporters at key barriers of interest^43,45,48,49^ and a haemochorial placental structure^50^. However, some differences do exist in absolute transporter levels and some aspects of placental morphology^43,45,48,49^. In addition, metabolism of different drugs differs between species.

Parallel studies in human placentas at different gestational ages, insofar as these are ethically possible, would contribute to validating results from rat studies. However, these were beyond the scope of the present study.

## Conclusions

As one of the most common drugs administered during pregnancy, understanding the conditions that determine paracetamol distribution in the fetus and particularly in its brain, is important for assessing safety of paracetamol taken during pregnancy. We have shown that transfer of paracetamol from blood to brain differs depending not only on age (mother, fetus, newborn) but also the dose and length of the treatment. We found that if clinical doses were taken over an extended period of time, concentration of paracetamol metabolites in maternal circulation increased and the amount in fetal circulation decreased compared to when only a single dose was administered. Differences in metabolite concentrations combined with changes to the expression of ABC efflux related transporters and enzymes (e.g. *abcc1, gstm2*) at barrier interfaces may explain changes to overall blood to brain permeability in both adults and fetuses. These results have implications for toxicologists and clinicians, given the extent of fetal exposure to paracetamol and its metabolites after maternal administration. The study also highlights the level of ABC transporter transcripts and related enzymes present in placental, blood–brain and blood-CSF interfaces and tests their dynamic regulation following either acute or chronic molecular exposure at different developmental stages. These results provide a general framework for understanding barrier restriction for different drugs and their metabolites and the underlying mechanistic reasons why permeability differences may be observed.

## Supplementary Information

Supplementary Table S1.

Supplementary Table S2.

Supplementary Table S3.

Supplementary Table S4.

Supplementary Table S5.

## Abbreviations

ABC: ATP-binding cassette
BCRP: Breast cancer resistant protein
CSF: Cerebrospinal fluid
E: Embryonic (note that by longstanding convention all gestational ages in rodents are referred to as embryonic, but in this study E19 is a fetal stage)
IL1β: Interleukin 1β cytokine
i.p.: Intraperitoneal
i.v.: Intravenous
MRP: Multidrug resistance protein
P: Postnatal
PGP: P-glycoprotein
RNA-Seq: RNA sequencing
SD: Standard deviation
